# Artificial intelligence software available for medical devices: surgical phase recognition in laparoscopic cholecystectomy

**DOI:** 10.1007/s00464-022-09160-7

**Published:** 2022-03-09

**Authors:** Ken’ichi Shinozuka, Sayaka Turuda, Atsuro Fujinaga, Hiroaki Nakanuma, Masahiro Kawamura, Yusuke Matsunobu, Yuki Tanaka, Toshiya Kamiyama, Kohei Ebe, Yuichi Endo, Tsuyoshi Etoh, Masafumi Inomata, Tatsushi Tokuyasu

**Affiliations:** 1 Faculty of Information Engineering, Department of Information and Systems Engineering, Fukuoka Institute of Technology, 1-30-1 Wajiro higashi, Higashi-ku, Fukuoka, Fukuoka 811-0295 Japan; 2 Faculty of Medicine, Department of Gastroenterological and Pediatric Surgery, Oita University, Oita, Japan; 3 Customer Solutions Development, Platform Technology, Olympus Technologies Asia, Olympus Corporation, Tokyo, Japan

**Keywords:** Image classification, Artificial intelligence, Phase recognition, Deep learning, Laparoscopic cholecystectomy, Surgical data science

## Abstract

**Background:**

Surgical process modeling automatically identifies surgical phases, and further improvement in recognition accuracy is expected with deep learning. Surgical tool or time series information has been used to improve the recognition accuracy of a model. However, it is difficult to collect this information continuously intraoperatively. The present study aimed to develop a deep convolution neural network (CNN) model that correctly identifies the surgical phase during laparoscopic cholecystectomy (LC).

**Methods:**

We divided LC into six surgical phases (P1–P6) and one redundant phase (P0). We prepared 115 LC videos and converted them to image frames at 3 fps. Three experienced doctors labeled the surgical phases in all image frames. Our deep CNN model was trained with 106 of the 115 annotation datasets and was evaluated with the remaining datasets. By depending on both the prediction probability and frequency for a certain period, we aimed for highly accurate surgical phase recognition in the operation room.

**Results:**

Nine full LC videos were converted into image frames and were fed to our deep CNN model. The average accuracy, precision, and recall were 0.970, 0.855, and 0.863, respectively.

**Conclusion:**

The deep learning CNN model in this study successfully identified both the six surgical phases and the redundant phase, P0, which may increase the versatility of the surgical process recognition model for clinical use. We believe that this model can be used in artificial intelligence for medical devices. The degree of recognition accuracy is expected to improve with developments in advanced deep learning algorithms.

Surgical process modeling (SPM) has various benefits owing to its ability to identify separate surgical phases. Furthermore, the possibilities of SPM will expand with the advanced image recognition of deep learning [1]. Recognition technology for surgical phases using deep learning has been used in a variety of cases; for instance, predicting an operation’s end time with an image of the surgical field, supporting surgeons’ intraoperative decision-making [1–3], indexing surgical videos in a database [4, 5], and assessing operation skill using videos. Notably, it is necessary that a deep learning model used in an operation room has high versatility for an unknown image. Recently, deep learning systems to assist surgeons’ decision-making have undergone remarkable developments [6], and the demands for surgical phase recognition techniques will increase in the near future.

In our research, surgical phase recognition based on deep learning has been applied to laparoscopic cholecystectomy [4, 7] and laparoscopic sigmoidectomy [8, 9]. In both cases, the technology recognized the surgical phases of each operation with high accuracy, especially the surgical tools, which were important factors to increase the degree of recognition accuracy [7]. In an operation with standardized procedures, the current treatment and pre- and posttreatment can be predicted from the surgical field and the type of surgical tool. Similarly, related studies [10, 11] have reported that surgical tools provide effective information to improve the recognition accuracy of the surgical phase. Importantly, in this method, using surgical tools to identify the surgical phase, the recognition accuracy often declines owing to different colors of the hook shaft of endoscopic instruments [12]. Additionally, blood on surgical tools and manipulations behind the organs are factors decreasing the recognition accuracy of the surgical phase. Furthermore, after upgrading the appearance of a surgical tool, we must reconstruct the learning model by repeating a series of development cycles, such as annotation, training, and evaluation. If the learning model has already been embedded in a commercially available medical device, the reconstructed learning model must undergo regulatory examination at each redesign related to the appearance of a surgical tool. Considering the cost involved in updating the learning model, accurately recognizing the surgical phase without relying on the information provided by surgical tools is important to predict the surgical phase.

EndoNet, proposed by Twinanda et al. [7] achieved approximately 0.82 overall accuracy for surgical phase recognition in laparoscopic cholecystectomy (LC), in which the features of an image from an endoscopic camera and of a surgical tool are used to predict the surgical phase. The authors used the open datasets Cholec80 and EndoVis, which contain the video data of LC performed in a single facility. Additionally, the authors adopted long short-term memory (LSTM) in a recurrent neural network to estimate the surgical phase while considering the surgical phase to a certain point, resulting in 0.963 recognition accuracy [5, 13]. Also, the authors proposed a deep learning model with LSTM to estimate the remaining surgery duration intraoperatively [14].

However, we considered that LSTM is not desirable to intraoperatively identify the surgical phase because unexpected intraoperative events happen frequently. In this regard, no redundant phase between the surgical phases was defined in either Cholec80 and EndoVis; [7, 15] therefore, the benefits of LSTM were limited in these datasets. With the development of the latest deep learning models, it has become possible to recognize the surgical process with high accuracy without using the recognition information of surgical instruments for decision-making [8, 16]. However, for extracorporeal images, misty images during sectioning, and out-of-focus images, it is difficult for even deep learning models to accurately estimate the surgical process from the information from a single image. Therefore, in addition to improving the accuracy of the learning model, postprocessing to estimate the surgical process is important for clinical use of the learning model. In this study, we aimed to construct a deep CNN model that intraoperatively identifies the surgical phase in LC and can be available as embedded software in a medical device. To accomplish our purpose, the surgical phase was recognized using only the endoscopic images obtained in LC.

LC was developed approximately 30 years ago and is now a standard treatment for benign pathologies, such as cholelithiasis and cholecystitis. The operative procedure for LC is mature and standardized, and is currently frequently implemented [17, 18]. Therefore, LC is often chosen in SPM research. The incidence of bile duct injury (BDI) during LC is an issue [19]. To prevent BDI, it has been strongly recommended to archive critical view of safety (CVS) data [12, 20] or to confirm the anatomical landmarks, such as the common bile duct or cystic duct, during the surgical phase of confirming Calot's triangle in the gallbladder neck [21, 22].

Recently, the use of a deep CNN model to reduce the incidence of BDI has been reported. Tokuyasu et al. [23] proposed an AI system that intraoperatively indicates the anatomical landmarks during confirming Calot's triangle; Mascagni et al. [24] proposed an automatic assessment tool for CVS during dissection of Calot's triangle; and Madani et al.[25] developed a deep learning model that visually identifies safe (Go) and dangerous (No-Go) zones for liver, gallbladder, and hepatocytic triangle dissection during LC. The purpose of these applications is limited to the specified surgical phase of confirming Calot’s triangle and Calot’s triangle dissection. We assume the surgical phase recognition model would be expected as a trigger for these applications.

In current LC, medical devices for confirming surgical field information include magnifying endoscopes, special light observation, such as narrow-band imaging and indocyanine green, and intraoperative ultrasonography. However, it is the surgeon who grasps the surgical field situation from the visual information presented by these medical devices. The surgeon need to use knowledge based on their own surgical experiences and the anatomical position of the organ to proceed with operation. Surgical field information misinterpretation by inexperienced surgeons may lead to an increased risk of intraoperative complications or an increase in surgical time. To address this issue, AI medical devices built on big data can objectively determine the status of the operation and present this information to the surgeon, which is important for improving the efficiency of the operation. In long operations, doctors as well as anesthesiologists, nurses, and other members of the team participate in the operation, and some members are replaced during the operation. Sharing information regarding the surgical phase recognition inside and outside the operating room will help the surgical team members understand the status of the operation and improve efficiency in the operating room. Our objective in this study was to assess the ability of a deep CNN model to identify the surgical phases during LC.

## Materials and methods

### Preparing the datasets

The videos of 115 cases of LC, after manually excluding cases with abnormal findings, such as fibrosis, scarring, and bleeding, were collected in this research. Cases with abnormal findings such as fibrosis, scarring, and hemorrhage are difficult to collect a sufficient number of cases for machine learning. These LC procedures were performed between January 2018 and January 2021. As a retrospective study, all operation videos were fully anonymized. This study was approved by the ethics committee of Oita University, Japan. Although the LC procedure can be divided into 10 surgical phases [16, 23], the action of confirming Calot's triangle and/or the running direction of the common bile duct were addressed in this study as a single surgical phase.

It is difficult to identify the doctor’s action when confirming the surgical field from image recognition. The LC surgical phases should comprise the actions involving physical intervention in a patient's organs, such as dissection and excision. Cholec80 is a major open dataset for the LC procedure, which comprises seven phases (P1–P7) [7]. Basically, our definition of a surgical phase was in accordance with Cholec80; however we integrated P5 (packing the gall bladder (GB)) and P7 (retrieving the GB) into one phase (GB retrieving). In addition, we included a redundant phase between actions considered a surgical process; for example, cleaning the endoscopic camera and changing endoscopic instruments. Finally, we defined the LC procedure by seven surgical phases, as described in Table 1.

**Table 1:** Definitions of the seven surgical phases (P0–P6)

Phase	Task	Start point/end point
P0	Other	Extracorporeal operation, trocar insertion, adhesiolysis, cleaning, other recovery, hemostasis, unexpected suture, drain insertion, trocar removal, etc.
P1	Preparation	Start: lifting gallbladder with grasping forceps
		End: completed clearance around the gallbladder
P2	Calot’s triangle dissection	Start: incising the gallbladder neck
		End: achieved critical view of safety
P3	Clipping and cutting	Start: inserting a clipping device to cut the cystic duct or artery
		End: completed cutting of the cystic duct or artery
P4	Gallbladder dissection	Start: dissecting gallbladder from the liver bed
		End: released gallbladder from the liver bed
P5	Gallbladder retrieving	Start: inserting retrieving bag
		End: removed the retrieving bag
P6	Cleaning and coagulation	Start: inserting a suction device
		End: removed the suction device

For this research, we created an original annotation tool that enabled adding the surgical phase label (P0–P6) to the video data by checking the image from the endoscopic camera. After finishing the annotations, the video data were converted into still images with the annotated label at 3 fps, and the data were saved to the computer. To evaluate the quality of our annotation dataset, two surgeons (both performed > 200 LC cases in over 10 years of surgical experience) performed the annotation, with consensus. In case of disagreement, a senior surgeon certified by the Japan Society of Endoscopic Surgery mediated.

We used 106 of the 115 annotated cases as the dataset to train our deep CNN model; 90 of 106 cases were used for training data, and the remaining 16 cases were used for validation. The training dataset was created by randomly extracting 10 000 images from each surgery in all 106 cases in the annotation datasets. The validation data were constructed in the same manner; however, the dataset for each surgical phase comprised 100 images. Finally, the remaining 9 of 115 cases were used to evaluate the trained deep CNN model.

### Deep learning model

Algorithms addressing the three major categories of deep learning, classification, semantic segmentation, and object detection, are updated daily. Many algorithms’ source codes are freely downloadable from the appropriate website. Algorithms for image classification, such as AlexNet and Inception-ResNet-v2, have been used frequently in studies of surgical phase recognition [7, 8]. In this study, we used EfficientNet [26] because its recognition accuracy assessed by general image datasets surpasses the performances of AlexNet and Inception-ResNet-v2. In addition, we also used the Sharpness-Aware Minimization (SAM) optimizer, which makes it possible to optimize the learning parameters of deep CNN models for image classification to smooth highly diverse image information for one label [27]. For the LC procedure, the appearance of an endoscopic image differs greatly between the start point and the end point of each surgical phase. We assumed that the combination of the latest deep learning techniques, EfficientNet and the SAM optimizer, have a higher possibility of achieving surgical phase recognition with high accuracy and versatility, especially for intraoperative use. In this research, EfficientNet-B7 was tentatively adopted as the base algorithm for our deep CNN model because this model has the highest accuracy among the EfficientNet series.

All work related to our deep CNN model was performed using a workstation (DGX Station V100; NVIDIA Corp., Santa Clara, CA).

### Evaluation of the deep CNN model

The nine LC videos for evaluating our deep CNN model (described in Sect. 2.1) and the evaluation dataset comprised 6300 images. Each surgical phase comprised 900 images randomly extracted from the nine LC videos. First, we evaluated the deep CNN model with the evaluation dataset and the metrics generally used in machine learning: accuracy, precision, and recall. These metrics can be described by the following equations ((1)–(3), below) [12, 13, 28], where TP is true positive, FP is false positive, FN is false negative, and TN is true negative:

1 $${\text{Accuracy}}\, = \,\left( {{\text{TP}}\, + \,{\text{TN}}} \right)/\left( {{\text{TP}}\, + \,{\text{FP}}\, + \,{\text{TN}}\, + \,{\text{FN}}} \right)$$

2 $${\text{Precision}}\, = \,{\text{TP}}/\left( {{\text{TP}}\, + \,{\text{FP}}} \right)$$

3 $${\text{Recall}}\, = \,{\text{TP}}/\left( {{\text{TP}}\, + \,{\text{FN}}} \right)$$

### Measures to address incorrect transition

According to our preliminary investigation, with the specification of our workstation, the average processing speed for one-time prediction of a surgical phase was 12 fps. Next, it is desirable to have a time interval for the processes of predicting a surgical phase. Because recognition accuracy is never 100%, a strategy was necessary to prevent incorrect transition of a surgical phase owing to misprediction of our deep CNN model. To address these issues, first, images from the LC video were input to the deep CNN model at 5 fps. To prevent incorrect transition of a surgical phase, we adopted the following measures: During periodic prediction of the surgical phase at 5 fps, if the probability of one-time prediction of the deep CNN model exceeded 80%, the predicted surgical phase was recorded as the candidate surgical phase. After 25 repetitions, if 24 or more of the same predictions were stocked, then the surgical phase was updated. Accordingly, the surgical phase updated every 5 s. The effect of this time lag of updating the surgical phase must be investigated through future verification testing. This measure to address misprediction of the deep CNN model in its online use may become an effective postprocessing method in the practical application of this model for surgical phase recognition.

## Results

### Statistics used to analyze the LC videos

**Fig. 1:**
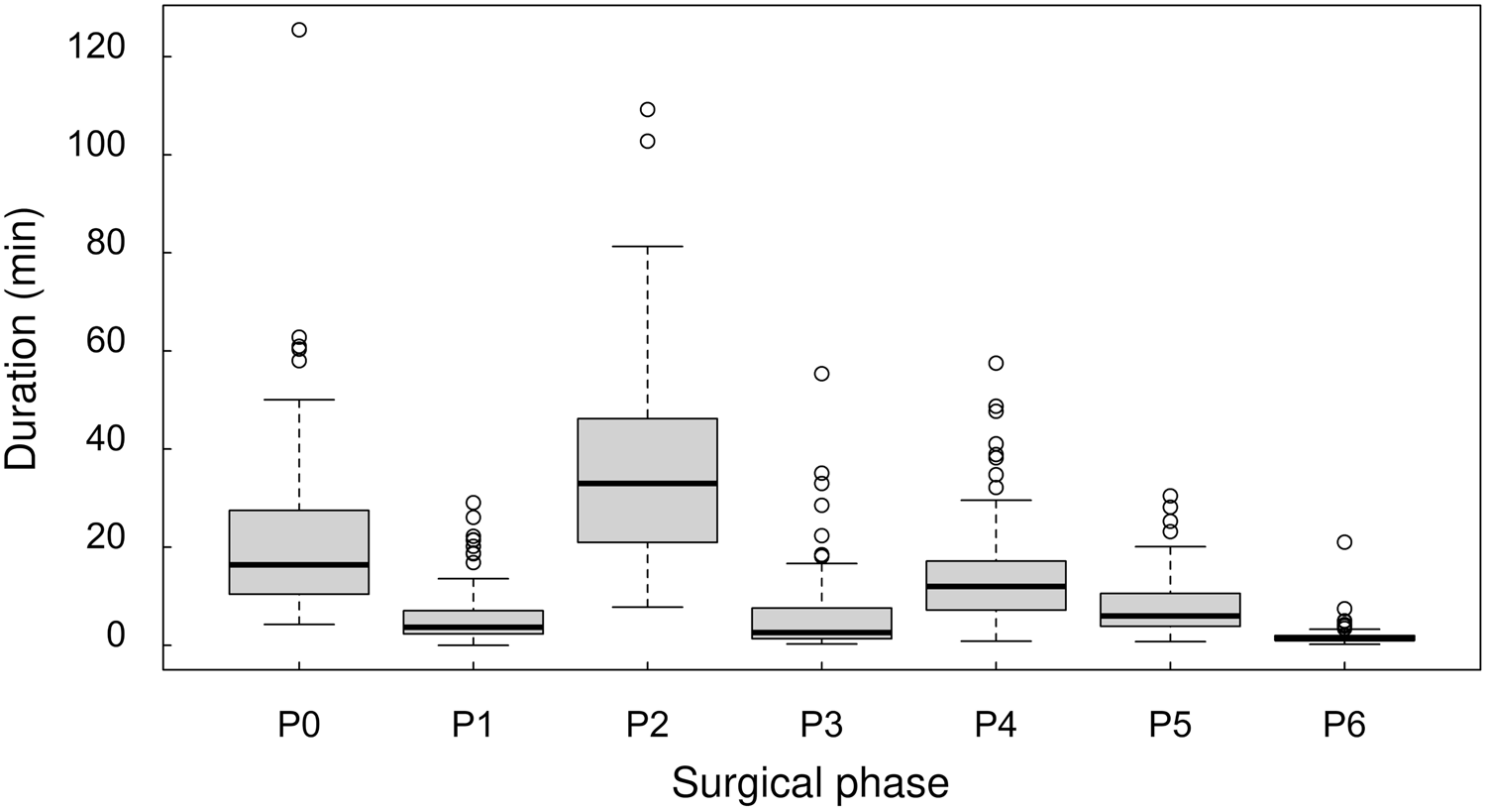
Analysis of the duration of the surgical phases in 115 LC cases. The duration differed for each phase and varied strongly between cases. The duration was 36.9 ± 19.8 min for P2, which was the longest surgical process, and 1.8 ± 2.1 min for P6, which was the shortest surgical process

**Fig. 2:**
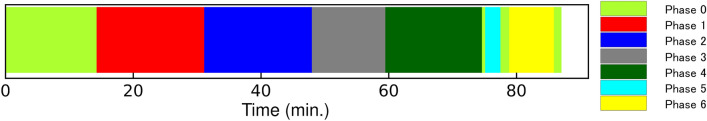
Schematic diagram of a representative transition in the surgical phase. The colors show each surgical phase. The horizontal axis of the color bar shows the time course of surgery, indicating the transition in surgical phase for each time point

The analyzed LC videos had an average surgical time of 94.0 min (standard deviation (SD): ± 41.6 min; *n* = 115). There was high variance in duration between cases, as described in Fig. 1. We used the full length of the video data, when all seven phases (P0–P6) were observed once or more in all videos. Basically, both the start phase and the end phase of the videos were considered P0 because the endoscopic camera is removed from the abdominal cavity, and this phase was considered a redundant phase in this study. The number of transitions of the surgical phase was 15.6 (SD: ± 5.4). In particular, the number of appearances of phase P0 was 5.6 (SD: ± 2.5). Figure 2 shows a representative case of surgical phase transition.

### Results of the surgical phase recognition evaluation

Table 2 shows the prediction results of our deep CNN model for all 6300 images from the evaluation dataset. The left column in Table 3 shows the results of converting the nine videos in the evaluation dataset into still images at 5 fps and continuously inputting the images into the deep CNN model. The overall accuracy, precision, and recall were 0.96 (SD: ± 0.04), 0.76 (SD: ± 0.21), and 0.85 (SD: ± 0.15), respectively.

**Table 2:** Results of the Offline Performance Test

		Prediction result (original) for each phase						
		P0	P1	P2	P3	P4	P5	P6
Ground truth	P0	677	40	30	15	39	25	74
	P1	49	663	168	2	6	8	4
	P2	7	22	770	30	54	4	13
	P3	9	1	93	719	42	4	32
	P4	5	1	16	7	848	9	14
	P5	80	7	2	0	38	737	36
	P6	46	0	4	2	7	9	832

**Table 3:** Recognized result with evaluation dataset

Surgical phase	Prediction result (original)			Prediction result (postprocessing)		
	Accuracy	Precision	Recall	Accuracy	Precision	Recall
P0	0.944	0.903	0.776	0.953	0.938	0.792
P1	0.965	0.779	0.759	0.975	0.830	0.837
P2	0.910	0.919	0.848	0.939	0.930	0.923
P3	0.975	0.586	0.824	0.986	0.745	0.772
P4	0.947	0.802	0.923	0.959	0.824	0.961
P5	0.988	0.507	0.853	0.994	0.802	0.807
P6	0.969	0.785	0.935	0.987	0.915	0.945

**Fig. 3:**
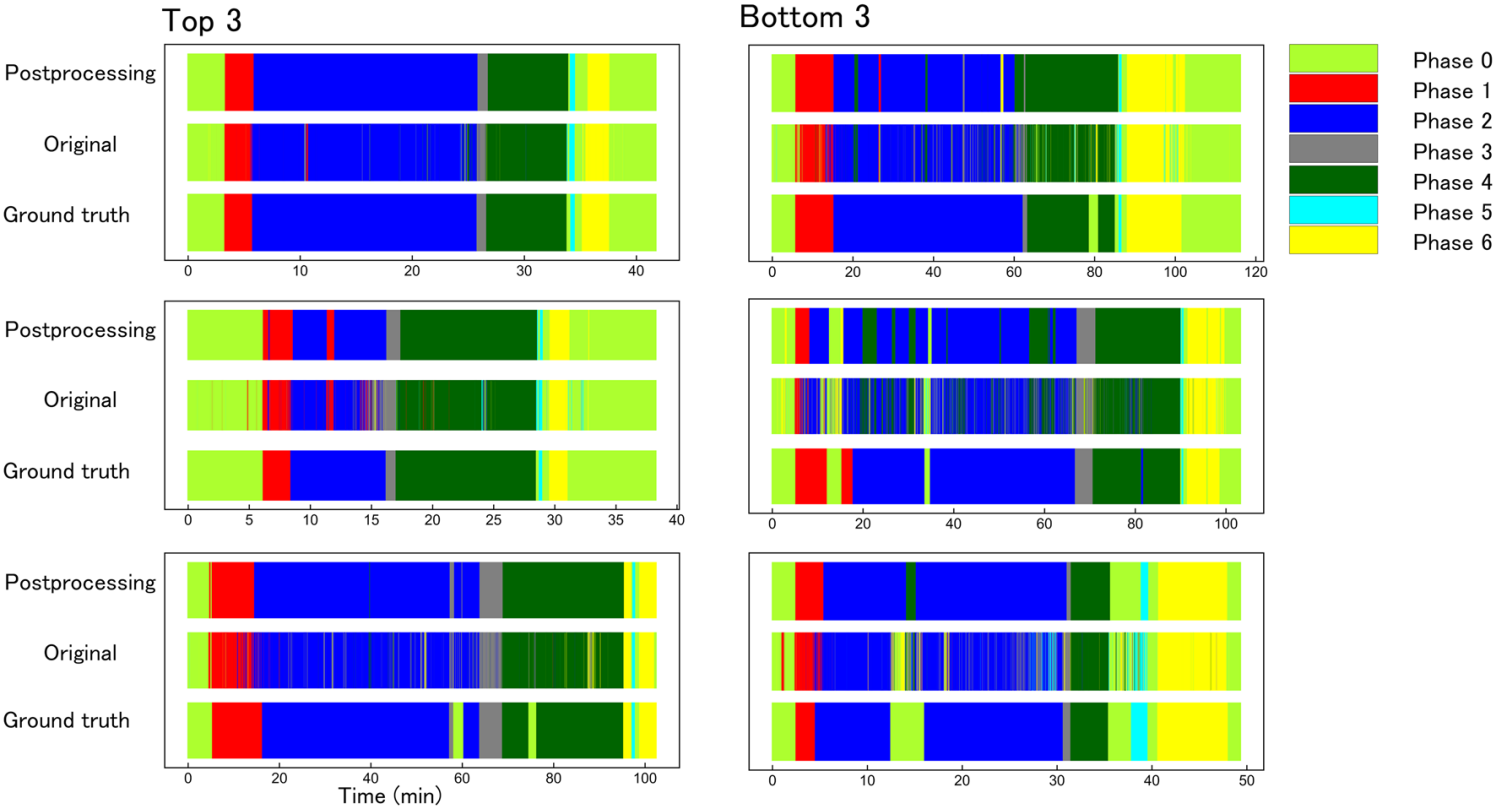
Transitions in surgical phases over time for ground truth, offline testing, and online testing. The left-hand column shows the order from the highest recall value in the nine test datasets. The right-hand column shows the order from the lowest recall value in the nine test datasets. The postprocessing results show inference by the artificial intelligence (AI) model, inference accuracy, and mode algorithm. The original results show inference from the AI model using EfficienteNet-B7 and SAM optimizer. The ground truth results show the time-dependent transition of the correct surgical process

The right column in Table 3 shows the results of converting the nine videos in the evaluation dataset to apply strategies to prevent accidental transitions in the surgical phase. The results of postprocessing against the misprediction of the surgical phase, as described in Sect. 2.4, are also shown in Fig. 3. Using these results, the average number of transitions of the surgical phase could be reduced from 966.4 (SD: ± 620.6) to 26.4 (SD: ± 12.8 SD).

## Discussion

In this study, the results using the deep learning model and postprocessing showed that average values were 0.97, 0.85, and 0.86 for overall accuracy, precision, and recall, respectively. The accuracy was higher than with the deep learning model alone, and flickering of the surgical process owing to false inference was suppressed.

The full-length LC videos we used in this study had an average surgical time of 94.0 min (SD: ± 41.6; *n* = 115), and the number of surgical phase transitions was 15.6 (SD: ± 5.4). Notably, the average surgical time in the videos contained in Cholec80 was 38.4 (SD: ± 17.0; *n* = 80) minutes, and the surgical phase transitioned 5.8 (SD: ± 0.39) times. Because Cholec80 was constructed as open datasets to stimulate research in SPM, the variation in the surgical difficulties in the LC videos might have been lower. In our assumption, there may be a redundant scene between the surgical phases, which was classified as surgical phase P0 in this study. However, the scene that we classified as the redundant phase was categorized as its previous surgical phase, uniformly, in Cholec80. We considered that the definition of the redundant phase (P0) is an essential factor to ensure that the deep CNN model keeps predicting the surgical phase stably intraoperatively because it is difficult to avoid dealing with extracorporeal images or to prevent an abnormal image owing to the visuality of the endoscopic camera. Most of the LC videos collected in this study were benign biliary tract diseases, and few had strong abnormal findings in acute cholecystitis. Although we intentionally excluded LC videos with many abnormal findings and poor anatomical visibility, all of the remaining videos showed average surgical difficulty, in accordance with usual difficulty in a university hospital. The AI learns to correlate the information in the image with the surgical process. However, if the features of abnormal findings are strongly reflected in the image, the AI cannot relate the findings to the surgical process. However, we have to consider the necessarily for making a surgical phase recognition model for high-difficulty cases such as acute cholecystitis in the future, that needs a sufficient number of LC data with abnormal findings.

In the present article, we successfully constructed a deep CNN model that achieved 0.970 overall accuracy for full-length LC videos. However, the metrics, precision and recall were approximately 0.700 for P3. As causes, we considered that the deep CNN model we trained tended to misunderstand phases P2 and P3, as shown in Table 2. There were many mistakes between the scene of incising the gallbladder neck (P2) and the scene of cystic duct cutting (P3). The reason for these mistakes is that AI mechanically classifies image classes from a single image feature, and it is difficult for AI to determine the surgeon’s intentions from a single image. We can determine the surgeon’s intent from the flow of work before and after as a video. The awareness of confirming the cystic duct and the gallbladder artery crosses phases P2 and P3, and the change of image features is not so different between the endo of P2 and the beginning of P3. This small difference limits the classification from the image features. In previous studies [7, 28], the accuracy of determining P2 and P3 was also low. However, there is need to improve the accuracy of detecting P2 and P3, which are important processes in LC, in future work.

Regarding the result for P0, owing to an out-of-focus image corresponding to improper operation of the endoscopic camera, and overexposed, enlarged, and misty images related to using energy devices, P0 was misjudged regardless of the surgical phase. These issues related to the visuality of the endoscopic image were also confirmed in similar research [29] and became the causes of incorrect transitions of the predicted surgical phase. Although some misjudgments can be dealt with by future hardware technological innovations, it is desirable to solve this issue by devising appropriate software.

Previous studies [4, 7, 13, 15, 29–31] used a variety of methods to improve the degree of accuracy in the online use of deep learning models, such as the recognition information of surgical tools, LSTM, and the hidden Markov model. Certainly, LSTM and the hidden Markov model are effective for LC videos that proceed according to a standardized operation procedure; in other words, the transition pattern of the surgical phase is fixed to some extent [5]. No surgeon accurately knows the situation even a few seconds ahead in an operation. The surgical phase changes in multiple patterns depending on the surgical situation; therefore, we decided to use the redundant phase, P0, and created a new simple postprocessing method for online use of a deep CNN model.

It has been widely recognized that archiving CVS data is a significant factor to avoid BDI during LC. Numerous studies have focused on the process of archiving CVS [23, 24, 32, 33] by developing technology in deep CNN models as AI software. In our experimental results, we determined that devising a model that correctly recognizes Calot’s triangle dissection would contribute to making the CVS archiving technology more practical.

AI software for clinical use has been developed worldwide since deep learning has become a common medical technology. Currently, the concept of “software as a medical device” (SaMD) has been discussed in pharmaceutical approval organizations, such as the Food and Drug Administration [34], Pharmaceuticals and Medical Devices Agency [35], and related medical societies [36]. To obtain pharmaceutical approval as an SaMD, we must verify the function of our deep CNN model using datasets obtained with informed patient consent for commercial use. This also applies to post-commercial updates of SaMD. Before commercialization of an SaMD, fewer software updates are required, to decrease development costs.

The considerable factors involved in updating an SaMD are the evolution of core software algorithms and expanding the scope of the target surgical level. Jin et al. [28] achieved 0.924 accuracy using SV-RCNet after reaching the limit of improving accuracy of a CNN algorithm. Regarding the evolution of core algorithms for image classification using deep learning, Inception-ResNet-v2 [8], Xception [16], and other custom CNN models [4] have progressively updated their worldwide accuracy. The SAM optimizer we used to train our deep CNN model using EfficientNet-B7 was introduced in 2020.

In our preliminary research, we constructed a CNN model using Inception-ResNet-v2 to predict the LC surgical phase using the same training dataset that we used in this paper. The results for the nine full-length videos resulted in postprocessing scores of 0.936, 0.679, and 0.689 for overall accuracy, precision, and recall, respectively. Using the SAM optimizer to train the CNN model using Inception-ResNet-v2, the results improved noticeably to 0.944, 0.719, and 0.705 for overall accuracy, precision, and recall, respectively.

Current AI technology could drastically improve the performance in a few months. However, the scope of the target surgical difficulty is a significant issue for the medical application of AI software. Abnormal findings owing to acute cholecystitis and excessive visceral fat decrease the visibility of anatomical structures. Deep CNN models rely on the image features of anatomical structures drawn on the input image. It is easy to say our results might have been worse if we did not exclude the LC videos of acute cholecystitis and excessive visceral fat. Although we did not discuss how to address expanding the scope of target difficulty in this paper, a solution is needed for this issue, to increase the practicality of our proposed method. It is generally believed that with surgical advances in endoscopic surgery, surgical processes such as laparoscopic sigmoidectomy [8, 37] are becoming increasingly standardized. If the surgical process can be clearly defined, as in Table 1, it is possible to create annotation data, and the proposed method in this paper can be applied. However, it may not be possible to obtain the high accuracy rate that we obtained for LC in this study because of patient factors, numerous changes in the order of surgical phases, and procedures with a small number of cases.

The sharing of objective information on the recognition of the surgical process by AI inside and outside the operating room facilitates communication among the members of the surgical team and also helps improve operating room efficiency. Previous studies [23–25] have evaluated AI to support the surgeon's judgment, and these AIs are now being used to support the surgeon's decision-making. By recognizing objective scenes, it will be possible to automatically switch AI on and off. In addition, considering the future of robotic surgical support, the robot can be expected to pass the appropriate surgical forceps to the surgeon based on the recognition information of the surgical process.

## Conclusions

In this study, we successfully established a deep learning CNN model that enables automatic identification of the surgical phase in full-length LC videos. In addition, we drastically reduced the number of incorrect transitions of the surgical phase owing to misidentification by the deep CNN model. The application of deep CNN technology to intraoperatively identify the surgical phase is expected to improve operating efficiency. For this technology to have sufficient functionality to be approved as a medical device, we will perform clinical performance tests and determine the issues that must be resolved for its practical clinical use.
